# PPARG as a central regulator of ferroptosis in Alzheimer’s disease: integrated transcriptomic, single-cell, and experimental evidence

**DOI:** 10.3389/fnagi.2026.1759279

**Published:** 2026-03-18

**Authors:** Lingjia Tang, Ningning Wu, Hong Xu, Yuxuan Mo

**Affiliations:** 1 Department of Geriatrics, Ningbo No.2 Hospital, Ningbo, Zhejiang, China; 2 Department of General Surgery, Ningbo No.2 Hospital, Ningbo, Zhejiang, China

**Keywords:** Alzheimer’s disease, ferroptosis, iron dyshomeostasis, lipid peroxidation, neurodegeneration, PPARG, scRNA-seq dataset

## Abstract

**Background:**

Alzheimer’s disease (AD) is a progressive neurodegenerative disorder characterized by cognitive decline, β-amyloid (Aβ) plaque accumulation, neurofibrillary tangle formation, and chronic neuroinflammation. Increasing evidence suggests that ferroptosis, an iron-dependent form of regulated cell death driven by lipid peroxidation, contributes to AD pathogenesis; however, its upstream regulatory mechanisms remain incompletely understood.

**Methods:**

Transcriptomic datasets from the Gene Expression Omnibus (GSE1297, GSE5281, and GSE157827) were analyzed to identify AD-associated differentially expressed genes (DEGs). Ferroptosis-related genes were obtained from curated databases and intersected with AD-associated DEGs. Protein–protein interaction (PPI) networks were constructed using the STRING database and analyzed via Cytoscape to identify key regulatory genes. Immune cell infiltration was quantified using CIBERSORT. Molecular docking was performed to evaluate ligand–PPARG binding. Functional validation was conducted using *in vitro* neuronal ferroptosis models and an *in vivo* AD mouse model.

**Results:**

A total of 50 reproducible differentially expressed genes (DEGs) were identified across two independent transcriptomic datasets, of which 24 genes were associated with ferroptosis. Network analysis consistently identified peroxisome proliferator-activated receptor gamma (PPARG) as a central hub gene within the ferroptosis regulatory network. Immune infiltration analysis revealed increased M2 macrophage abundance in AD tissues, suggesting an association between ferroptosis-related gene expression and immune-related transcriptional signatures. Molecular docking demonstrated stable ligand binding to PPARG with a binding affinity of −5.6 kcal/mol, supported by hydrogen-bond and hydrophobic interactions. Experimental validation confirmed that PPARG modulation significantly influenced ferroptosis-associated neuronal injury both *in vitro* and *in vivo*.

**Conclusion:**

These findings identify PPARG as a key regulator linking ferroptosis and neuroinflammation in Alzheimer’s disease. Targeting PPARG-mediated ferroptotic pathways may therefore represent a promising therapeutic strategy for mitigating neurodegeneration in AD.

## Introduction

Alzheimer’s disease (AD) is a progressive neurodegenerative disorder and the leading cause of dementia worldwide, characterized by cognitive decline, memory impairment, and loss of functional independence. AD represents a growing global health challenge, with approximately 50 million individuals affected worldwide in 2020, a number projected to exceed 150 million by 2050 due to population aging (Zeisel et al., 2020). Despite decades of intensive research, current therapeutic strategies provide only modest symptomatic relief, and effective disease-modifying treatments remain limited (Long and Holtzman, 2019; Cummings et al., 2024; Lane et al., 2018). This highlights the urgent need to identify novel molecular mechanisms underlying AD pathogenesis and to develop innovative therapeutic targets.

The pathological hallmarks of AD include extracellular β-amyloid (Aβ) plaque deposition, intracellular neurofibrillary tangles composed of hyperphosphorylated tau, synaptic loss, and chronic neuroinflammation (Selkoe and Hardy, 2016; Heneka et al., 2015). Although the amyloid and tau hypotheses have dominated the field, it is increasingly recognized that AD is a multifactorial disorder involving metabolic dysfunction, oxidative stress, iron dyshomeostasis, and immune dysregulation (De Strooper and Karran, 2016; Wang et al., 2022; Ma et al., 2022). Importantly, these pathological processes are not independent but interact dynamically to exacerbate neuronal vulnerability and disease progression.

Ferroptosis is a distinct, regulated form of cell death driven by iron-dependent lipid peroxidation and overwhelming oxidative stress, mechanistically distinct from apoptosis, necroptosis, and pyroptosis (Dixon et al., 2012; Stockwell et al., 2017). The execution of ferroptosis is governed by tightly regulated networks controlling iron metabolism, lipid remodeling, and antioxidant defenses, including glutathione peroxidase 4 (GPX4), the cystine–glutamate antiporter system Xc^−^ (SLC7A11), and enzymes involved in polyunsaturated fatty acid metabolism (Yang and Stockwell, 2016). Neurons are particularly susceptible to ferroptosis due to their high metabolic demand, abundant lipid content, and limited antioxidant capacity (Do Van et al., 2016).

Growing evidence implicates ferroptosis as a key contributor to AD pathophysiology. Postmortem and neuroimaging studies consistently demonstrate abnormal iron accumulation in vulnerable AD brain regions, including the hippocampus and cerebral cortex, correlating with cognitive decline and neurodegeneration (Raven et al., 2013; Tran et al., 2022; Bao et al., 2021). Iron overload promotes Aβ aggregation, facilitates tau hyperphosphorylation, and amplifies oxidative stress through Fenton chemistry (Belaidi and Bush, 2016; Ashraf et al., 2018). In parallel, lipid peroxidation products generated during ferroptosis act as damage-associated molecular patterns, driving microglial activation and perpetuating neuroinflammation (Friedmann Angeli et al., 2019; Li et al., 2020). Notably, genetic or pharmacological inhibition of ferroptosis has been shown to alleviate neuronal loss and cognitive deficits in experimental models of AD, supporting ferroptosis as a therapeutically relevant mechanism (Bao et al., 2021; Li et al., 2023; Zhong et al., 2024).

Peroxisome proliferator-activated receptor gamma (PPARG) is a ligand-activated nuclear receptor that plays a central role in lipid metabolism, redox homeostasis, and inflammatory regulation (Berger and Moller, 2002; Evans and Mangelsdorf, 2014). PPARG signaling has been widely implicated in neuroprotection, primarily through anti-inflammatory and metabolic mechanisms (Kapadia et al., 2008; Heneka et al., 2005). Emerging studies suggest that PPARG may also modulate ferroptotic vulnerability by regulating lipid peroxidation pathways, iron handling, and antioxidant systems, including crosstalk with the NRF2–GPX4 axis (Yapryntseva et al., 2022; Wu et al., 2024; Su et al., 2019). However, PPARG signaling is highly context-dependent, and recent evidence indicates that its functional consequences vary across cell types and pathological states (Lehrke and Lazar, 2005; Ahmadian et al., 2013). In the context of AD, where neurons are exposed to chronic oxidative stress and iron accumulation, the precise role of PPARG in ferroptosis regulation remains poorly defined.

Recent advances in single-cell transcriptomics have further revealed profound cell-type-specific transcriptional reprogramming in AD, particularly within neuronal populations (Mathys et al., 2019; Grubman et al., 2019; Leng et al., 2021). These technologies provide unprecedented resolution to dissect disease-related regulatory pathways and identify cell-type-restricted therapeutic targets. Integrating ferroptosis biology with single-cell transcriptomic data may therefore offer critical insights into neuronal vulnerability in AD.

In this study, we systematically investigated the molecular relationship between ferroptosis and Alzheimer’s disease, with a specific focus on PPARG as a potential regulatory node. By integrating bulk transcriptomic analyses, protein–protein interaction network construction, functional enrichment, immune infiltration profiling, and scRNA-seq, we identified key ferroptosis-related genes involved in AD pathogenesis. Molecular docking, *in vitro* ferroptosis assays, and *in vivo* validation further clarified the functional role of PPARG. Collectively, our findings identify PPARG as a central regulator linking ferroptosis, metabolic dysregulation, and neuroinflammation in AD, providing new mechanistic insight and highlighting a potential therapeutic target for ferroptosis-driven neurodegeneration.

## Materials and methods

### Data sources and preprocessing

Transcriptomic data for Alzheimer’s disease (AD) were obtained from two publicly available Gene Expression Omnibus (GEO) datasets: GSE1297, comprising 22 AD and 9 control samples, and GSE5281, including 87 AD and 74 control samples. Raw expression values were log₂-transformed following the addition of a pseudocount of 1 to prevent undefined values. Probes without corresponding gene annotations were excluded. Remaining probes were mapped to gene symbols according to the respective microarray platforms, and for genes represented by multiple probes, expression values were averaged to generate a single representative value.

### Differential expression analysis

Differential expression analysis between Alzheimer’s disease (AD) and control samples was performed independently for the GSE1297 and GSE5281 datasets using the limma package. Raw *p* values were adjusted for multiple testing using the Benjamini–Hochberg false discovery rate (BH-FDR) method. Genes with BH-FDR < 0.05 and |log₂ fold change (log₂FC)| > 1 were defined as differentially expressed genes (DEGs) and retained for subsequent analyses.

### Identification of ferroptosis-related genes

To identify ferroptosis-associated genes involved in AD, DEGs were intersected with curated ferroptosis-related gene sets (including drivers, suppressors, and markers) obtained from the FerrDb database.^1^ Overlapping genes were visualized using Venn diagrams generated with the *VennDiagram* package (version 1.7.3).

### Protein–protein interaction network construction

Protein–protein interaction (PPI) networks for ferroptosis-related DEGs were constructed using the STRING database.^2^ Only interactions with a confidence score greater than 0.4 were retained. The resulting PPI networks were visualized and analyzed using Cytoscape software (version 3.10.0).

### Functional enrichment and pathway analysis

Functional annotation of ferroptosis-related DEGs was performed using the *clusterProfiler* package in Bioconductor. Gene Ontology (GO) enrichment analysis was conducted to identify enriched biological processes, cellular components, and molecular functions, using the human genome as the background reference. Kyoto Encyclopedia of Genes and Genomes (KEGG) pathway enrichment analysis was also performed. Enriched GO terms and pathways were identified using a significance threshold of *p* < 0.05, with a minimum gene count of ≥2 for GO terms and ≥3 for KEGG pathways. Enrichment results were visualized using dot plots.

### Immune cell infiltration analysis

Relative immune cell-type fractions were estimated using the CIBERSORT algorithm implemented via the IOBR package (v0.99.0). We used the LM22 signature matrix and performed permutations. Deconvolution was run on the batch-corrected, normalized expression matrix restricted to genes present in the signature. Samples were retained for downstream analyses only if the CIBERSORT deconvolution *p* value < 0.05, consistent with recommended quality filtering. Immune fractions were interpreted as relative signature enrichments rather than absolute cell counts.

### Cell lines and culture conditions

PC-12 and SH-SY5Y cells were obtained from the Chinese Academy of Sciences Cell Bank (Shanghai, China) and were authenticated by short tandem repeat (STR) profiling. Routine mycoplasma testing was performed prior to experimentation. PC-12 cells were cultured in RPMI-1640 medium supplemented with 10% horse serum, 5% fetal bovine serum (FBS), and 1% penicillin–streptomycin, while SH-SY5Y cells were cultured in DMEM/F12 medium supplemented with 10% FBS and 1% penicillin–streptomycin. All cells were maintained at 37 °C in a humidified incubator with 5% CO₂.

Cells were used within passages 5–15 for all experiments. SH-SY5Y cells were used in an undifferentiated state, as no retinoic acid–induced neuronal differentiation was applied unless otherwise stated.

### Reagents and treatment conditions

Ferroptosis was induced using RSL-3 (0.5 μM) or erastin (20 μM) as described in the Results section of the manuscript. The PPARG inhibitor FTX-6746 was dissolved in dimethyl sulfoxide (DMSO) and used at a final concentration of 10 μM. For co-treatment experiments, cells were pre-treated with FTX-6746 for 2 h, followed by exposure to RSL-3 or erastin for 24 h, unless otherwise specified. In selected experiments, FTX-6746 was administered concurrently with ferroptosis inducers, yielding comparable results.

The final DMSO concentration was maintained at 0.1% (v/v) in all experimental groups, including vehicle controls. Cells were seeded at a density of 2 × 10^5^ cells/well in 6-well plates (or 5 × 10^3^ cells/well in 96-well plates for viability assays) and treated at approximately 70–80% confluence.

### Experimental groups (*in vitro*)

The following experimental groups were included in all *in vitro* assays:

Control (vehicle only; 0.1% DMSO)

FTX-6746 alone (10 μM)

RSL-3 alone (0.5 μM)

RSL-3 + FTX-6746

Erastin alone (20 μM)

Erastin + FTX-6746

All experiments were performed with at least three independent biological replicates.

### Ligand selection rationale and docking controls

Molecular docking was performed to evaluate binding feasibility between PPARG and candidate lipid ligands. 10,12-octadecadienoic acid was selected because it is a representative polyunsaturated fatty-acid ligand relevant to lipid peroxidation / ferroptosis-linked lipid remodeling. To strengthen inference, we included control docking runs: (i) a known PPARG ligand as a positive control (e.g., rosiglitazone/pioglitazone), and (ii) a structurally related lipid or randomized ligand as a negative control.

### Brain-specific validation and robustness checks

Because LM22 was originally derived from peripheral immune populations and bulk brain transcriptomes can be confounded by cellular composition, we performed an orthogonal validation using a brain-relevant approach. Specifically, we quantified microglia/myeloid signature scores using established microglia-enriched marker genes (e.g., TMEM119, P2RY12, AIF1) and compared these scores with CIBERSORT-inferred myeloid/M2-like signatures. In addition, we performed a brain cell-type deconvolution using [BRETIGEA/scRNA-seq reference (GSE157827)] to estimate major brain cell classes and myeloid enrichment.

### Identification of hub genes

Key hub genes within the ferroptosis-related PPI network were identified using the CytoHubba plug-in in Cytoscape. Three topological algorithms—Degree, Betweenness, and maximal clique centrality (MCC)—were applied to rank genes according to their network importance. Genes consistently ranked highly across these methods were considered hub genes.

### scRNA-seq dataset

scRNA-seq from the GSE157827 dataset were analyzed to investigate cell-type–specific expression patterns in AD. Data preprocessing, normalization, and clustering were performed using the Seurat package (version 5.1.0), with a clustering resolution set to 0.3. Dimensionality reduction was conducted using Uniform Manifold Approximation and Projection (UMAP). Cell-type annotation was performed using the SingleR package (version 2.0.0), followed by refinement through ScType-assisted annotation and manual curation based on established marker genes reported in the literature. Marker gene expression across cell types was visualized using violin plots. The expression distribution and differential patterns of PPARG between AD and control groups were examined using feature plots.

### Measurement of lipid peroxidation and cytosolic ROS

Lipid peroxidation and cytosolic reactive oxygen species (ROS) levels were measured using a lipid peroxidation sensor and CM-H2DCFDA, respectively, according to the manufacturers’ instructions. Briefly, cells were incubated with the respective fluorescent probes in culture medium at 37 °C under 5% CO₂ for 30 min. Following incubation, cells were washed with phosphate-buffered saline (PBS) and analyzed by flow cytometry within 2 h of staining.

### Cellular iron staining

Intracellular iron levels were assessed using Phen Green™ SK staining. Cells were washed twice with PBS and incubated with 10 nM Phen Green™ SK at 37 °C for 15 min. After staining, cells were centrifuged, resuspended in PBS, and analyzed using a BD Fortessa X30 flow cytometer. Changes in fluorescence intensity were interpreted as inversely proportional to intracellular iron levels.

### Establishment of Alzheimer’s disease mouse model

Six-week-old BALB/c mice (Beijing Vital River Laboratory Animal Technology Co., China) were used to establish an AD model. AD-like phenotypes were induced through long-term systemic administration of D-galactose. All procedures followed the guidelines of the Laboratory Animal Centre of Zhejiang Chinese Medical University (approval number: IACUC-20230924-05).

### Batch-effect correction and dataset integration

To enable combined downstream immune deconvolution analyses across independent transcriptomic cohorts, GSE1297 and GSE5281 were merged after preprocessing and gene symbol mapping. Only genes shared between datasets were retained. Expression values were log₂-transformed (log₂[x + 1]) and standardized to ensure comparable scaling across platforms. Batch effects attributable to dataset origin were corrected using the ComBat algorithm implemented in the sva package, with dataset (GSE1297 vs. GSE5281) specified as the batch variable. To preserve biological differences of interest, the model matrix included diagnosis (AD vs. control) as a covariate. Default parametric adjustments were used, and corrected expression matrices were subsequently used as input for CIBERSORT-based immune deconvolution.

### Quality control and validation of batch correction

Batch correction efficacy was evaluated using multiple complementary approaches: (i) PCA before and after ComBat correction to visualize dataset mixing and separation by diagnosis; (ii) distribution-based QC using density plots and boxplots (and RLE plots) to confirm alignment of expression distributions across datasets; and (iii) quantitative variance partitioning using PVCA (or an equivalent variance decomposition approach) to estimate the proportion of expression variance attributable to dataset/batch and diagnosis before vs. after correction. All QC plots and PVCA results are provided in Supplementary Table S5.

### PARG target engagement and ferroptosis marker assessment

To establish mechanistic directionality and on-target engagement of PPARG inhibition, canonical PPARG target genes (e.g., CD36, FABP4, PLIN2, CPT1A/ACOX1, SCD) were quantified by qPCR and/or immunoblotting following FTX-6746 treatment under basal and ferroptosis-inducing conditions. Core ferroptosis markers were assessed, including GPX4 and SLC7A11 (system Xc−), ACSL4, TFRC and FTH1, intracellular GSH levels, and lipid peroxidation endpoints (MDA and/or 4-HNE). These readouts were analyzed alongside functional ferroptosis phenotypes (iron, ROS, lipid ROS) to connect PPARG inhibition to ferroptosis pathway modulation.

### Assessment of brain ferroptosis markers

Following behavioral testing, mice were euthanized and hippocampal tissues were collected for biochemical and histological analyses. Iron accumulation was assessed using Perls’ Prussian blue staining on paraffin-embedded brain sections and quantified by measuring total iron content using a colorimetric iron assay kit according to the manufacturer’s instructions.

Lipid peroxidation was evaluated by measuring malondialdehyde (MDA) levels using a thiobarbituric acid–reactive substances (TBARS) assay and/or by immunohistochemical detection of 4-hydroxynonenal (4-HNE) adducts. All quantitative analyses were performed by investigators blinded to group allocation.

### Selection of ferroptosis-related genes

Ferroptosis-related genes were obtained from the FerrDb database, which provides a curated collection of genes experimentally validated as drivers, suppressors, or markers of ferroptosis. Unlike Gene Ontology (GO) pathways, which often include broad and heterogeneous gene sets associated with oxidative stress or lipid metabolism, FerrDb focuses specifically on genes with direct mechanistic evidence linking them to ferroptotic cell death. This curated framework was therefore used to prioritize biologically specific ferroptosis regulators for downstream analyses.

### Dataset context and pathological heterogeneity

The bulk transcriptomic datasets analyzed in this study were derived from postmortem human brain tissue and encompass different anatomical regions and levels of pathological characterization. GSE1297 focuses exclusively on the hippocampus, whereas GSE5281 includes multiple cortical and limbic regions that exhibit differential vulnerability during neurodegeneration. Although donors were classified as Alzheimer’s disease or control based on clinical and neuropathological evaluation, detailed staging information (e.g., Braak or CERAD scores) was not uniformly reported in the original GEO submissions. Consequently, disease stage–specific analyses were not feasible. This heterogeneity represents an inherent limitation of public postmortem datasets and may contribute to inter-sample variability.

### Donor-aware statistical testing for scRNA-seq

To statistically compare PPARG expression between AD and control groups while accounting for donor-level dependence, we performed pseudobulk differential expression analysis. Briefly, within each annotated cell type, we aggregated raw counts across cells per donor to generate a pseudobulk count matrix (cell type × donor). Pseudobulk counts were normalized (e.g., TMM/size factors) and analyzed using a generalized linear model framework (e.g., edgeR/DESeq2 or limma-voom), with diagnosis (AD vs. control) as the explanatory variable and donor as the unit of replication.

For each cell type, we report PPARG log₂ fold change (AD vs. control), raw *p* value, and Benjamini–Hochberg FDR (BH-FDR). In addition, we computed a standardized effect size (Cohen’s d) based on donor-level normalized expression values to quantify the magnitude of change. *p* values across tested cell types were adjusted using BH-FDR.

### Molecular docking analysis

Molecular docking was performed to explore the structural feasibility of interactions between peroxisome proliferator-activated receptor gamma (PPARG) and lipid ligands relevant to ferroptosis-associated lipid metabolism. The crystal structure of human PPARG ligand-binding domain was obtained from the Protein Data Bank, and protein preparation included removal of water molecules, addition of polar hydrogens, and assignment of partial charges.

Known PPARG ligands include endogenous fatty acids and eicosanoids (e.g., polyunsaturated fatty acids), as well as synthetic agonists such as thiazolidinediones (e.g., rosiglitazone and pioglitazone), which validate the lipid-binding capacity of PPARG. Based on this established lipid-binding profile, we constructed a candidate ligand pool enriched for ferroptosis-relevant lipid species, including polyunsaturated fatty acids and lipid peroxidation-associated molecules.

Candidate selection strategy.

Candidate ligands were selected according to the following criteria:

- documented association with lipid metabolism or oxidative stress;
- relevance to ferroptosis-related lipid remodeling; and
- chemical compatibility with the PPARG ligand-binding pocket.

### Docking procedure

Docking simulations were performed with a grid box centered on the canonical ligand-binding pocket. Binding affinity scores and interaction residues were analyzed to assess binding feasibility. A known PPARG ligand (e.g., rosiglitazone) was included as a positive control, and a structurally related lipid was used as a negative control to contextualize docking scores.

### Statistical analysis

All experiments were performed with at least three independent biological replicates. Statistical analyses were conducted using GraphPad Prism software. Data are presented as mean ± standard error of the mean (SEM). Comparisons between two groups were performed using Student’s *t* test or the Wilcoxon rank-sum test, while multiple-group comparisons were analyzed using one-way analysis of variance (ANOVA). A *p* value less than 0.05 was considered statistically significant.

### Ethics approval and consent to participate

All animal experiments were approved by the Institutional Animal Care and Use Committee (IACUC) vide (approval number: IACUC-20230924-05) and were conducted in accordance with the National Institutes of Health Guide for the Care and Use of Laboratory Animals. No human participants were involved in this study.

## Results

### Transcriptomic identification of Alzheimer’s disease–associated genes

In the GSE1297 dataset, [N1] DEGs were identified between AD and control samples, whereas [N2] DEGs were detected in the GSE5281 dataset using BH-FDR < 0.05 and |log₂FC| > 1 as significance thresholds. To identify reproducible AD-associated transcriptional changes, we intersected DEG lists from both datasets and retained genes exhibiting concordant directions of differential expression, yielding 50 shared DEGs.

Intersection of these 50 reproducible DEGs with curated ferroptosis-related gene sets from FerrDb identified 24 ferroptosis-associated genes, which were subsequently subjected to network, immune infiltration, single-cell, and experimental analyses.

**Figure 1:**
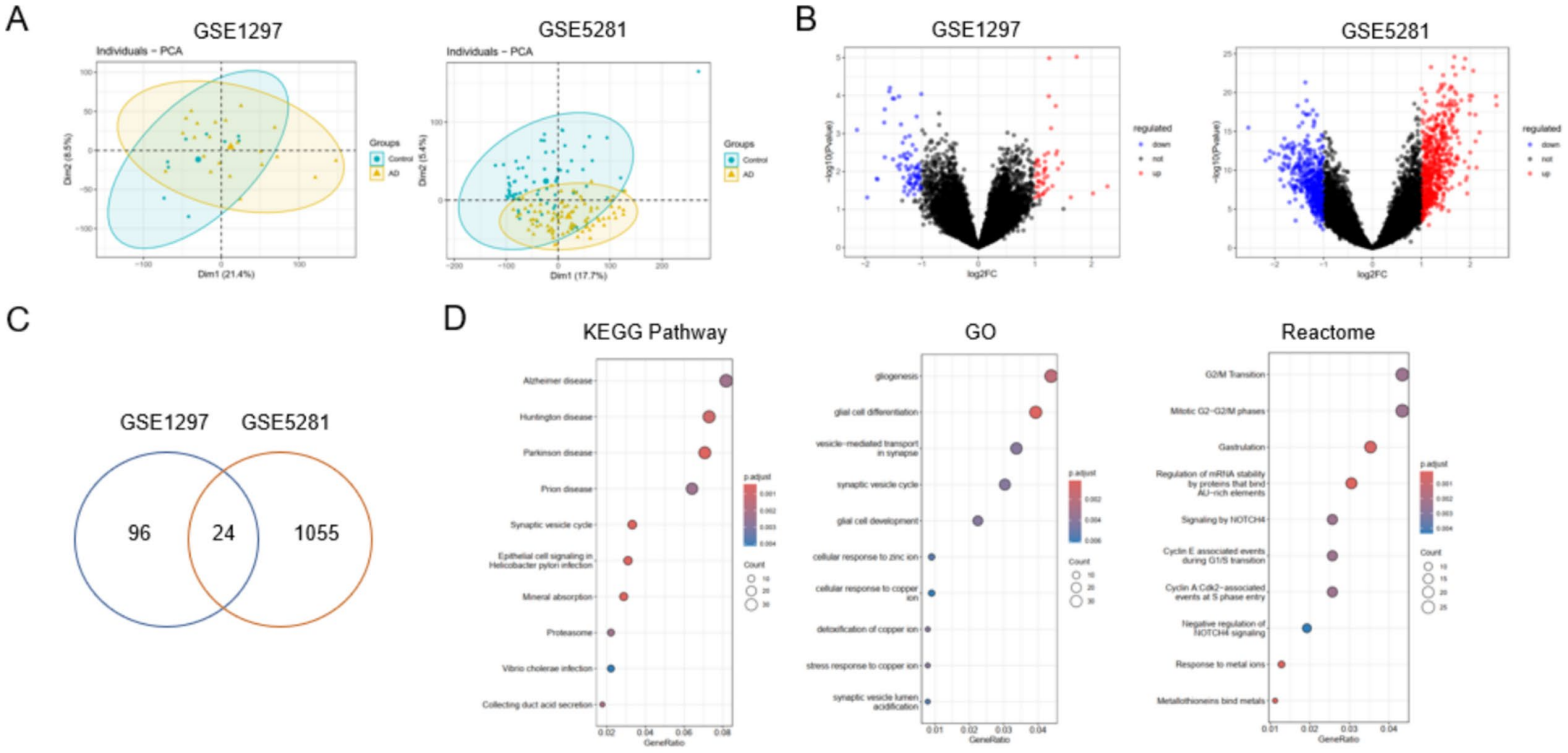
Transcriptomic identification of Alzheimer’s disease–associated genes. Transcriptomic analysis identifies Alzheimer’s disease–associated differentially expressed genes. **(A)** Principal component analysis (PCA) demonstrating clear global transcriptomic separation between Alzheimer’s disease (AD) and control samples in the GSE1297 and GSE5281 datasets. **(B)** Volcano plots showing differentially expressed genes (DEGs) between AD and control groups in each dataset; red and blue dots represent significantly upregulated and downregulated genes, respectively. **(C)** Venn diagram illustrating the overlap of DEGs shared between the two datasets. **(D)** Kyoto encyclopedia of genes and genomes (KEGG), gene ontology (GO), and reactome pathway enrichment analyses highlighting biological processes and signaling pathways associated with AD-related DEGs.

To characterize transcriptional alterations associated with Alzheimer’s disease (AD), two independent microarray datasets (GSE1297 and GSE5281) were analyzed. PCA revealed partial separation with substantial overlap between AD and control samples, indicating that disease status was not the sole driver of transcriptomic variance (Figure 1A). Differential expression analysis identified a robust set of differentially expressed genes (DEGs) in AD compared with controls, including both significantly upregulated and downregulated genes (Figure 1B).

Intersection analysis demonstrated a substantial overlap of DEGs between the two datasets, supporting the reproducibility of AD-associated transcriptional changes (Figure 1C). It illustrates the overlap of differentially expressed genes (DEGs) identified independently in GSE1297 and GSE5281 using consistent statistical thresholds. The complete list of overlapping DEGs is provided in Supplementary Table Sx. Among these overlapping genes, a subset annotated as ferroptosis-related in FerrDb was selected for focused analysis and is summarized in Table 1. Functional enrichment analyses using Gene Ontology (GO), Kyoto Encyclopedia of Genes and Genomes (KEGG), and Reactome databases revealed that these DEGs were significantly enriched in pathways related to metabolic regulation, redox homeostasis, synaptic signaling, immune responses, and cell death processes (Figure 1D). These findings indicate that dysregulated metabolic and redox pathways, together with immune-related mechanisms, are central features of AD transcriptomic alterations.

**Table 1:** Ferroptosis-related differentially expressed genes implicated in AD.

Gene	Ferroptosis role	Regulation in AD	Functional annotation
ACSF2	Driver	Up	Fatty-acid metabolism
GOT1	Driver	Up	Amino-acid metabolism
GABARAPL1	Driver	Up	Autophagy regulation
ELAVL1	Driver	Up	RNA stability
EPAS1	Driver	Up	Hypoxia signaling
POR	Driver	Up	Redox metabolism
SNCA	Driver	Up	Synaptic function
SIRT3	Driver	Down	Mitochondrial redox control
WWTR1	Driver	Up	Hippo pathway signaling
IFNA8	Driver	Up	Immune response regulation
PPARG	Suppressor	Up	Lipid metabolism, ferroptosis regulation
KLF2	Suppressor	Down	Transcriptional regulation
GJA1	Suppressor	Up	Gap junction communication
PGRMC1	Suppressor	Up	Steroid signaling
CFL1	Suppressor	Down	Actin cytoskeleton
RB1	Suppressor	Down	Cell cycle control
MT1G	Suppressor	Up	Metal ion binding
SLC40A1	Suppressor	Up	Iron export
SCD	Suppressor	Down	Lipid desaturation
LAMP2	Marker	Down	Lysosomal function
PARP9	Driver	Up	DNA repair
PARP11	Driver	Up	Immune signaling
CAMKK2	Driver	Down	AMPK signaling
RARRES2	Driver	Up	Chemoattractant activity

### Identification and functional characterization of ferroptosis-related genes in AD

**Figure 2:**
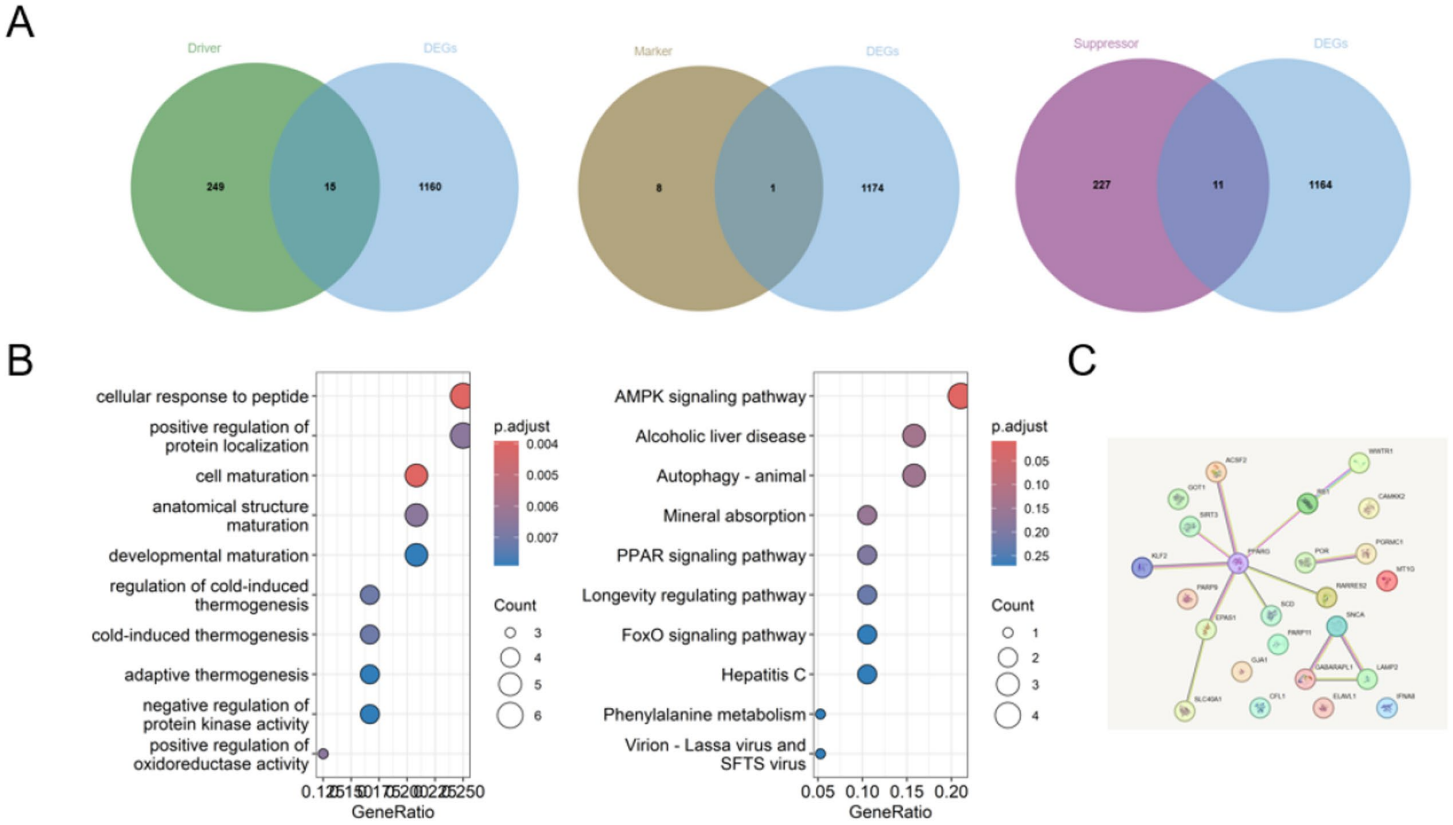
Identification and network characterization of ferroptosis-associated genes in Alzheimer’s disease. Integrated analysis identifies ferroptosis-associated genes involved in Alzheimer’s disease. **(A)** Venn diagram illustrating the intersection between AD-associated differentially expressed genes (DEGs) and ferroptosis-related genes categorized as drivers, suppressors, and markers. **(B)** Gene ontology (GO), Kyoto encyclopedia of genes and genomes (KEGG), and Reactome pathway enrichment analyses of ferroptosis-associated DEGs, highlighting significant biological processes and signaling pathways related to ferroptosis and metabolic regulation. **(C)** Protein–protein interaction (PPI) network of ferroptosis-associated genes constructed using the STRING database and visualized with Cytoscape, revealing close functional associations among key regulatory nodes.

Given the emerging role of ferroptosis in neurodegeneration, we next focused on identifying ferroptosis-related DEGs in AD. By intersecting AD-associated DEGs with curated ferroptosis driver, suppressor, and marker gene sets, we identified 24 ferroptosis-related genes significantly dysregulated in AD (Figure 2A). These genes displayed diverse functional roles encompassing lipid metabolism, redox regulation, mitochondrial function, autophagy, immune signaling, and iron homeostasis (Table 1).

Several ferroptosis drivers, including ACSF2, GOT1, GABARAPL1, EPAS1, SNCA, PARP9, PARP11, and RARRES2, were upregulated in AD, suggesting enhanced susceptibility to ferroptotic stress. In contrast, genes involved in mitochondrial redox control and cellular energy regulation, such as SIRT3 and CAMKK2, were downregulated. Notably, multiple ferroptosis suppressors, including PPARG, GJA1, PGRMC1, MT1G, and SLC40A1, exhibited increased expression, possibly reflecting compensatory mechanisms in response to lipid peroxidation and iron dysregulation.

Pathway enrichment analysis of ferroptosis-related DEGs revealed significant enrichment in metabolic pathways, hypoxia signaling, immune-related processes, and lipid regulatory networks (Figure 2B). Construction of a protein–protein interaction (PPI) network further demonstrated extensive connectivity among these genes, highlighting coordinated regulatory interactions rather than isolated molecular effects (Figure 2C).

### Immune landscape and ferroptosis–immune interactions in AD

**Figure 3:**
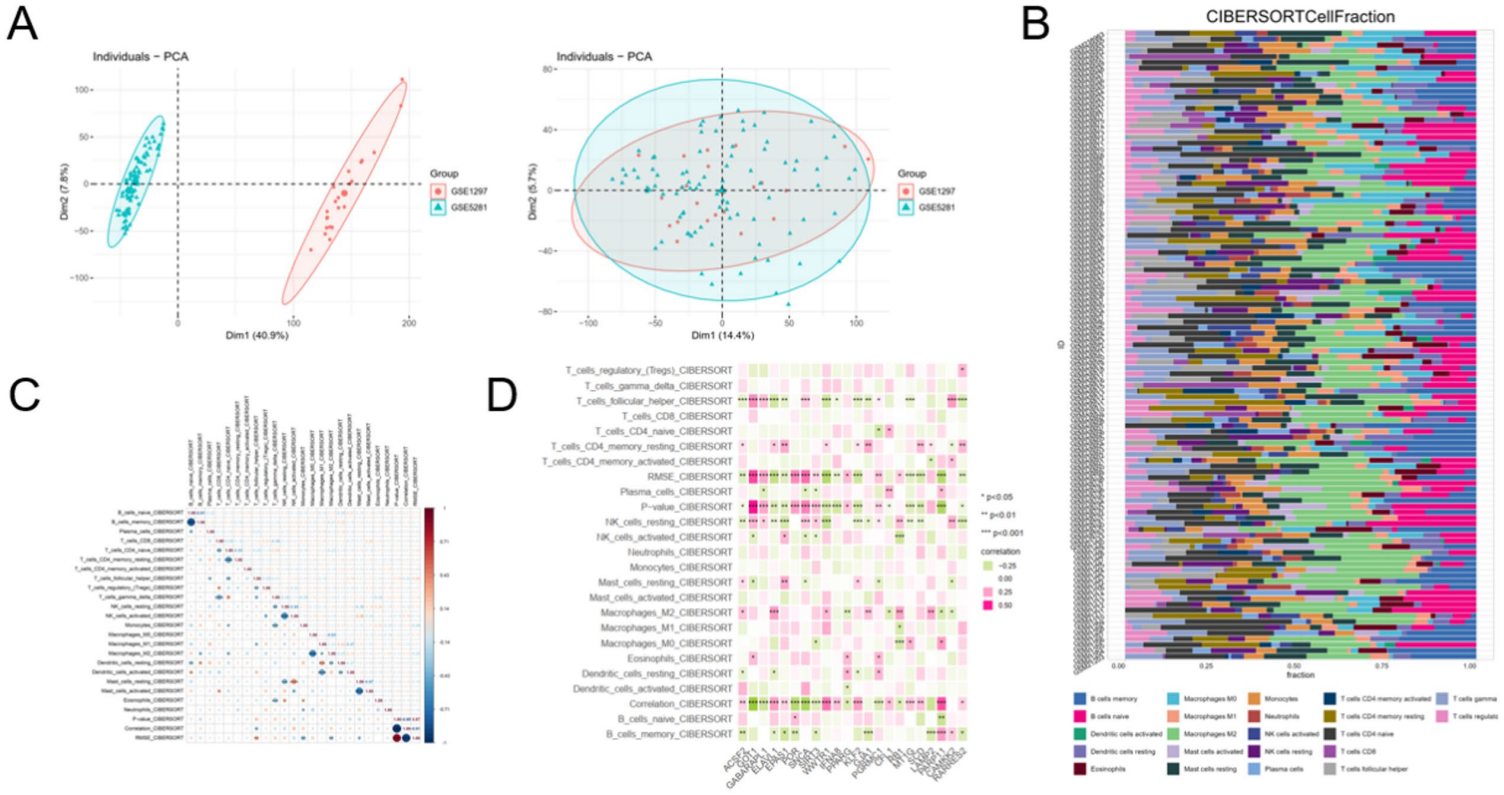
Immune landscape and ferroptosis–immune interactions in Alzheimer’s disease. Immune cell infiltration patterns and their associations with ferroptosis-related genes in Alzheimer’s disease. **(A)** Principal component analysis demonstrating effective batch effect removal and successful integration of the GSE1297 and GSE5281 datasets. **(B)** Relative immune signature proportions inferred by CIBERSORT (LM22) from bulk brain transcriptomic data. The *x*-axis represents relative contributions of immune gene-expression signatures, not absolute immune cell abundance. In brain tissue, these values should be interpreted as relative transcriptional similarities rather than direct measurements of immune cell infiltration. **(C)** Correlation matrix illustrating interactions between distinct immune cell populations within the AD microenvironment. **(D)** Heatmap depicting correlations between ferroptosis-related differentially expressed genes and immune cell infiltration levels, highlighting immune–ferroptosis associations in AD.

To investigate whether ferroptosis-related genes are associated with immune alterations in AD, Immune cell infiltration analysis was performed after correcting for dataset-origin batch effects using ComBat while preserving diagnosis as a covariate. PCA and distribution-based QC demonstrated improved cross-dataset integration after correction. Quantitative variance partitioning further confirmed that the variance attributable to dataset/batch was substantially reduced after correction (Supplementary Table S5), supporting the suitability of the corrected matrix for CIBERSORT-based immune deconvolution. PCA confirmed successful integration of the datasets with minimal technical bias (Figure 3A). Immune deconvolution revealed altered relative immune signature enrichments, particularly for myeloid-like programs, indicating a remodeled immune microenvironment (Figure 3B). Because bulk brain tissue contains limited numbers of peripheral immune cells, the inferred immune fractions primarily reflect relative enrichment of immune-associated transcriptional programs, with macrophage/microglia-like signatures being the most biologically plausible contributors.

Correlation analysis demonstrated complex interactions among immune cell populations (Figure 3C). Importantly, ferroptosis-related DEGs exhibited significant correlations with specific immune cell subsets (Figure 3D). In particular, PPARG expression showed positive associations with M2 macrophages, resting CD4 memory T cells, and T follicular helper cells (Supplementary Table S1), suggesting a potential interplay between PPARG-mediated ferroptosis regulation and immune modulation in AD.

### Network-based identification of PPARG as a hub gene and structural evaluation

**Figure 4:**
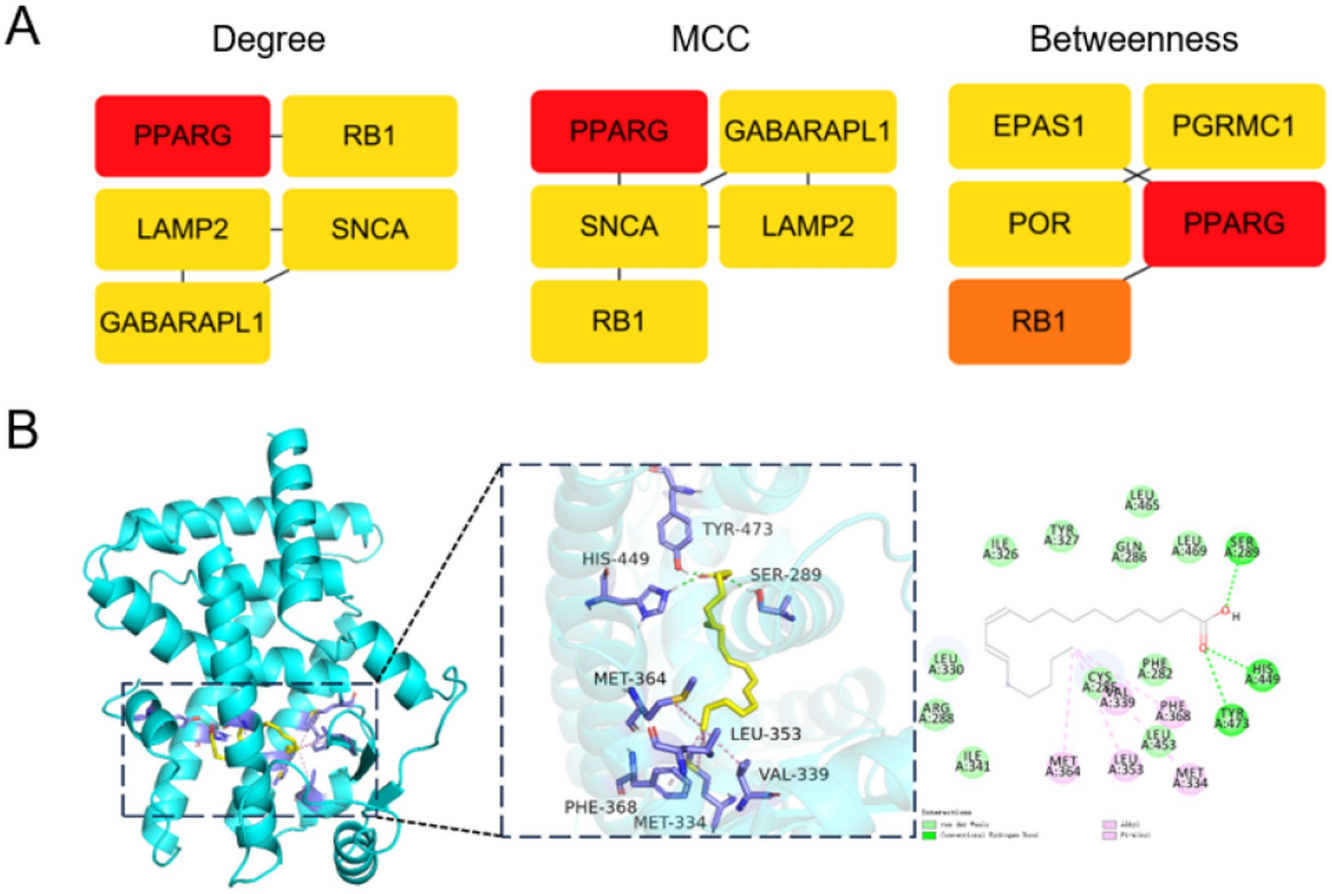
Hub gene identification and structural evaluation of PPARG as a therapeutic target. Network-based identification of hub genes and molecular docking analysis of PPARG. **(A)** Hub genes were ranked using three topological algorithms—Degree, maximal clique centrality (MCC), and Betweenness—applied to the ferroptosis-related protein–protein interaction network, consistently identifying PPARG as the top-ranked hub gene. **(B)** Molecular docking analysis showing the binding conformation of 10,12-octadecadienoic acid within the ligand-binding pocket of PPARG. The magnified view highlights hydrogen-bond interactions and hydrophobic contacts between the ligand and key amino acid residues, supporting the structural feasibility of ligand–PPARG interaction.

To identify key regulatory genes within the ferroptosis-associated network, we applied multiple network topology algorithms, including Degree, maximal clique centrality (MCC), and Betweenness centrality. Across all metrics, PPARG consistently ranked as a top hub gene, indicating a central regulatory role within the ferroptosis-related network (Figure 4A).

To further explore the functional relevance of PPARG, molecular docking analysis was conducted. Docking simulations demonstrated a stable binding conformation of 10,12-octadecadienoic acid within the ligand-binding pocket of PPARG, stabilized by multiple hydrogen-bond and hydrophobic interactions with key residues (Figure 4B). These findings provide structural support for the potential modulation of PPARG activity.

### scRNA-seq reveals neuronal enrichment of PPARG expression

**Figure 5:**
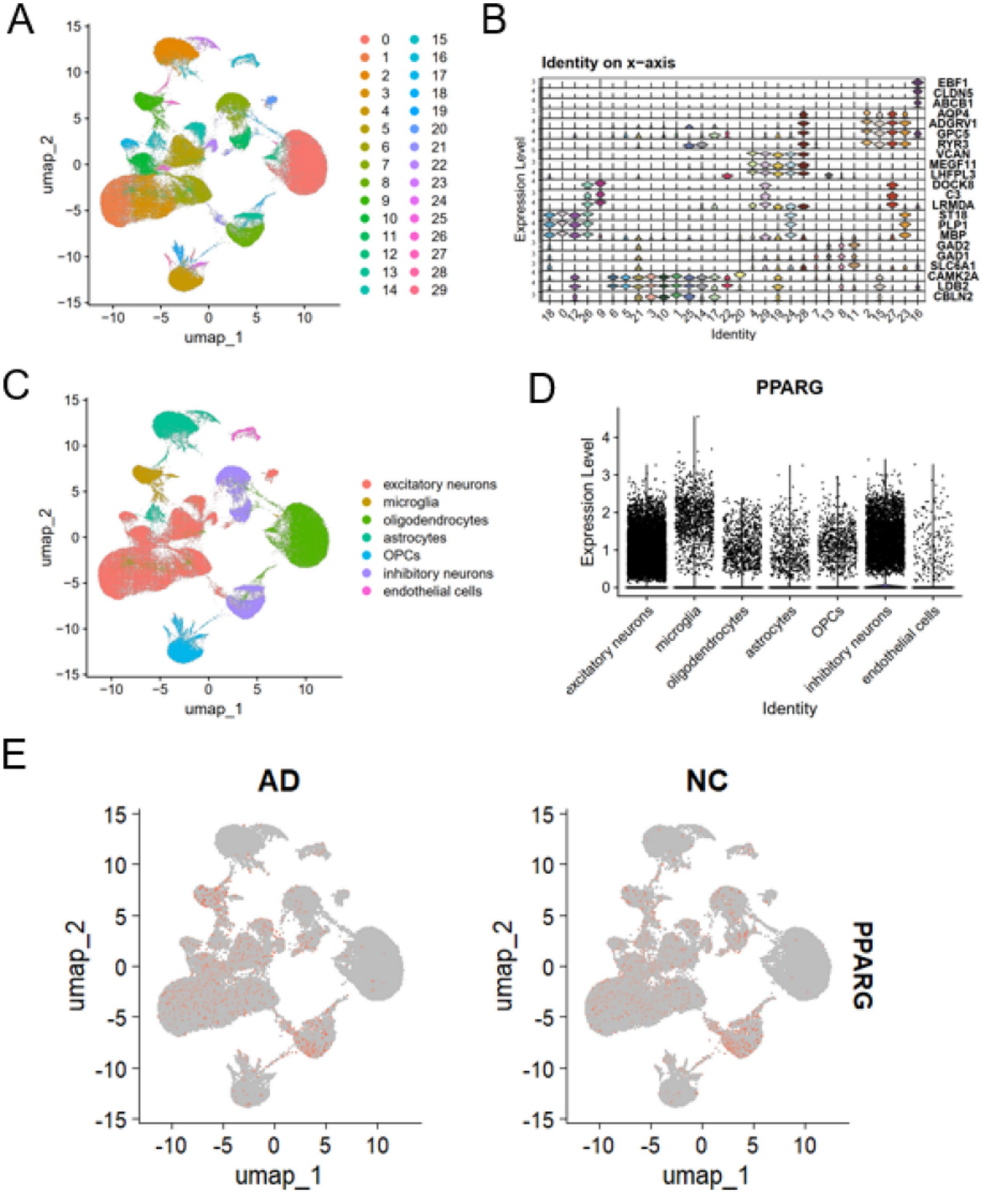
scRNA-seq reveals neuronal enrichment of PPARG expression in Alzheimer’s disease. Cell-type–specific expression patterns of PPARG in Alzheimer’s disease revealed by scRNA-seq. **(A)** Uniform manifold approximation and projection (UMAP) visualization showing 30 transcriptionally distinct cell clusters identified from the integrated scRNA-seq. **(B)** Dot plot illustrating the expression of canonical marker genes used for cluster annotation. **(C)** UMAP projection depicting the annotation of seven major brain cell types, including excitatory neurons, inhibitory neurons, astrocytes, microglia, oligodendrocytes, oligodendrocyte progenitor cells (OPCs), and endothelial cells. **(D)** Violin plot showing PPARG expression levels across the seven major cell types. **(E)** Side-by-side violin plots showing PPARG expression levels in each cell cluster derived from single-nuclei RNA-seq data. For each cluster, PPARG expression in AD and control samples is shown separately, enabling direct comparison of distribution and central tendency between conditions.

To determine the cell-type specificity of PPARG expression, scRNA-seq were analyzed. Unsupervised clustering identified 30 transcriptionally distinct clusters, visualized by uniform manifold approximation and projection (UMAP) (Figure 5A). Annotation based on canonical marker genes identified seven major brain cell types, including excitatory neurons, inhibitory neurons, astrocytes, microglia, oligodendrocytes, oligodendrocyte progenitor cells (OPCs), and endothelial cells (Figures 5B,C).

Analysis of PPARG expression across cell types revealed marked neuronal enrichment. Both excitatory and inhibitory neurons exhibited significantly increased PPARG expression in AD compared with controls, whereas astrocytes, oligodendrocytes, OPCs, and endothelial cells showed no significant changes. Microglia displayed only a mild increase in PPARG expression (Figures 5D,E; Table 2). These results indicate that PPARG dysregulation in AD is predominantly localized to neuronal populations.

**Table 2:** Cell-type–specific expression patterns of PPARG in Alzheimer’s disease revealed by scRNA-seq.

Cell type	PPARG expression in AD vs control
Excitatory neurons	Significantly increased
Inhibitory neurons	Significantly increased
Astrocytes	No significant change
Microglia	Mild increase
Oligodendrocytes	No significant change
Endothelial cells	No significant change
OPCs	No significant change

To statistically validate the cell-type–specific PPARG differences suggested by feature plots, we performed donor-aware pseudobulk testing within each annotated cell type. PPARG was significantly increased in excitatory neurons and inhibitory neurons in AD compared with controls (BH-FDR < 0.05), with moderate-to-large standardized effect sizes (Cohen’s d). In contrast, PPARG differences were not significant (BH-FDR ≥ 0.05) in astrocytes, oligodendrocytes, OPCs, and endothelial cells, while microglia showed a small increase that did not remain significant after multiple-testing correction (Supplementary Figure S1).

### Brain sectioning and anatomical alignment

For histological analyses, brains were fixed, paraffin-embedded, and coronally sectioned. To ensure anatomical comparability, hippocampal sections were selected from the same anterior–posterior plane across animals, corresponding to approximately bregma −1.8 to −2.2 mm, based on a standard mouse brain atlas. All staining and quantification were performed on matched sections by investigators blinded to experimental group allocation.

### PPARG inhibition suppresses neuronal ferroptosis *in vitro*

**Figure 6:**
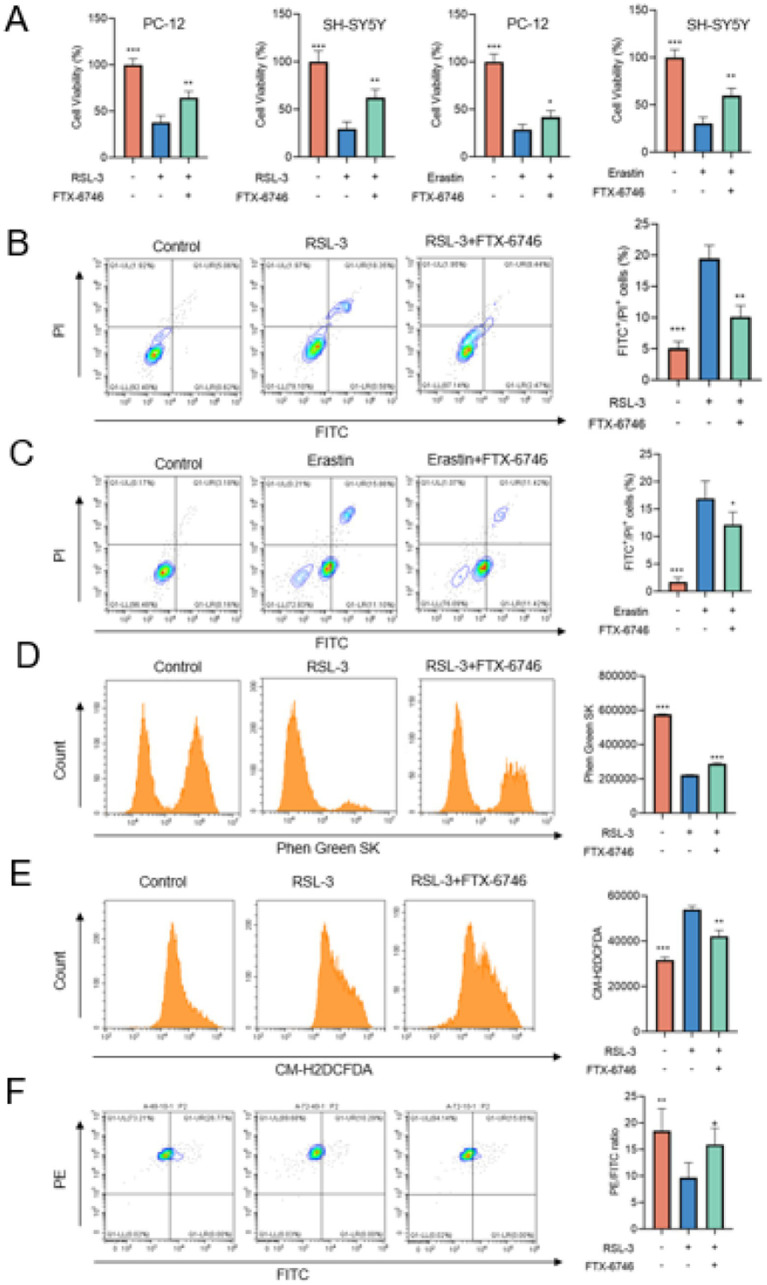
Functional inhibition of PPARG attenuates neuronal ferroptosis *in vitro*. PPARG inhibition protects neuronal cells against ferroptosis induced by RSL-3 and erastin. **(A)** Cell viability of PC-12 and SH-SY5Y cells treated with RSL-3 (0.5 μM) or erastin (20 μM) in the presence or absence of the PPARG inhibitor FTX-6746, demonstrating improved viability upon PPARG inhibition. (B, C) Flow cytometry analysis of ferroptotic cell death in PC-12 **(B)** and SH-SY5Y **(C)** cells using FITC/PI staining under RSL-3 or erastin treatment, showing reduced ferroptotic population following FTX-6746 co-treatment. **(D)** Phen Green SK–based quantification of intracellular iron levels, revealing increased iron accumulation after RSL-3 exposure and significant reversal upon PPARG inhibition. **(E)** Flow cytometric measurement of cytosolic reactive oxygen species (ROS) using CM-H_2_DCFDA, showing elevated ROS during ferroptosis and marked suppression by FTX-6746. **(F)** Lipid peroxidation assessment using a ratiometric lipid ROS sensor (PE → FITC), demonstrating ferroptosis-induced lipid peroxidation and its attenuation following PPARG inhibition.

To experimentally validate the role of PPARG in ferroptosis, we assessed the effects of PPARG inhibition in neuronal cell models. Exposure of PC-12 and SH-SY5Y cells to the ferroptosis inducers RSL-3 or erastin significantly reduced cell viability, consistent with ferroptotic cell death. Co-treatment with the PPARG inhibitor FTX-6746 markedly restored cell viability in both cell lines (Figure 6A).

Flow cytometric analysis confirmed that ferroptotic cell populations induced by RSL-3 or erastin were significantly reduced following PPARG inhibition (Figures 6B,C). Furthermore, intracellular iron accumulation, cytosolic reactive oxygen species (ROS) levels, and lipid peroxidation were all elevated during ferroptosis and significantly attenuated by FTX-6746 treatment (Figures 6D–F; Supplementary Table S2). These findings suggest that PPARG inhibition attenuates key molecular features associated with neuronal ferroptosis *in vitro*.

### FTX-6746 attenuates brain ferroptosis-associated alterations *in vivo*

To directly assess ferroptosis-related changes at the brain level, we evaluated iron accumulation and lipid peroxidation in hippocampal tissues. D-galactose–treated mice exhibited pronounced iron deposition, as evidenced by increased Perls’ Prussian blue staining and elevated total iron content compared with controls. In parallel, levels of lipid peroxidation markers, including malondialdehyde (MDA) and/or 4-hydroxynonenal (4-HNE), were significantly increased in the hippocampus, consistent with enhanced ferroptotic stress.

Importantly, FTX-6746 treatment markedly reduced iron accumulation and lipid peroxidation, restoring these ferroptosis-associated markers toward control levels. These findings provide direct *in vivo* evidence that PPARG inhibition mitigates ferroptosis-related brain injury in the D-galactose–induced model.

### Mechanistic directionality and context dependence of PPARG in neuronal ferroptosis

Although FerrDb categorizes PPARG as a ferroptosis “suppressor” based on evidence aggregated across diverse biological contexts, this classification does not necessarily predict PPARG function in neurons under AD-relevant oxidative stress. In our neuronal models, PPARG inhibition by FTX-6746 consistently reduced iron accumulation, ROS, and lipid peroxidation and improved viability following RSL-3/erastin challenge, supporting a functional role of PPARG signaling in promoting ferroptosis-associated injury in this context. Therefore, we interpret PPARG dysregulation in AD as context-dependent, potentially reflecting maladaptive metabolic and lipid remodeling that increases ferroptotic vulnerability.

### PPARG inhibition ameliorates cognitive impairment and hippocampal pathology *in vivo*

The neuroprotective effects of PPARG inhibition were next evaluated in an AD mouse model. Barnes maze testing revealed significantly increased escape latency in AD model mice, indicative of cognitive impairment. Treatment with FTX-6746 significantly reduced escape latency, indicating improved spatial learning and memory.

Open field testing showed that AD model mice exhibited increased locomotor activity, reflected by elevated travel distance and movement speed. These behavioral abnormalities were markedly reduced following PPARG inhibition. Histological analysis further revealed pronounced neuronal loss and tissue disorganization in the hippocampus of AD model mice, whereas FTX-6746 treatment preserved hippocampal architecture and reduced pathological damage (Supplementary Table S3).

### Integrative framework identifies PPARG as a central regulator of ferroptosis in AD

**Figure 7:**
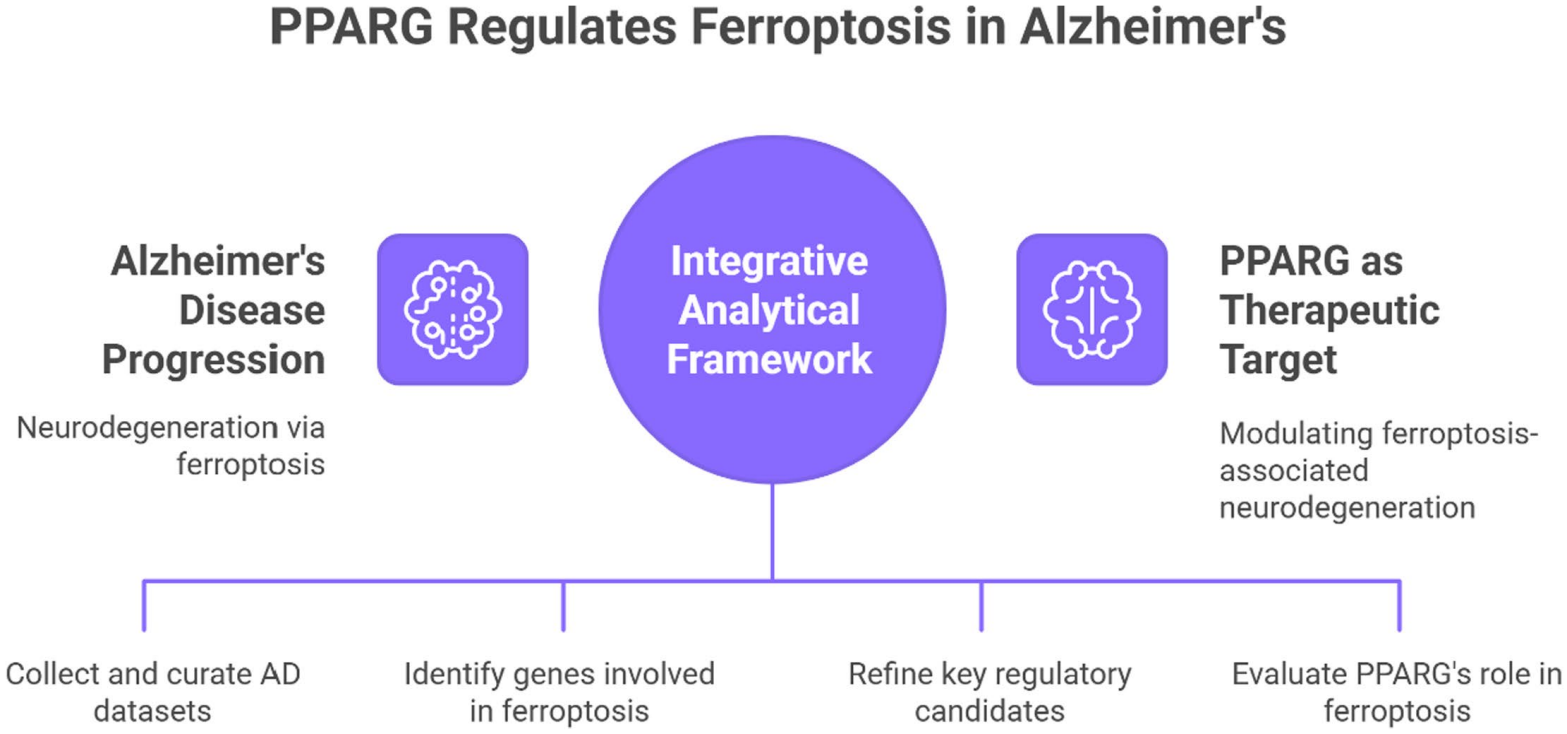
Integrative analytical framework identifying PPARG-mediated ferroptosis in Alzheimer’s disease.

Finally, transcriptomic, immune, single-cell, network, and experimental findings were integrated into a unified analytical framework (Figure 7). This framework highlights PPARG as a central regulator linking ferroptosis, metabolic dysregulation, and neuroinflammation in Alzheimer’s disease.

## Discussion

Alzheimer’s disease (AD) is a multifactorial neurodegenerative disorder characterized by progressive cognitive decline, synaptic dysfunction, and neuronal loss, driven by complex interactions between amyloid pathology, tau aggregation, neuroinflammation, and metabolic dysregulation (Long and Holtzman, 2019; Lane et al., 2018; Selkoe and Hardy, 2016; Heneka et al., 2015; De Strooper and Karran, 2016). Despite extensive efforts, disease-modifying therapies remain limited, highlighting the need to identify novel pathogenic mechanisms and therapeutic targets (Cummings et al., 2024). In this study, we provide integrative evidence that ferroptosis contributes to AD pathogenesis and identify peroxisome proliferator-activated receptor gamma (PPARG) as a critical regulator linking ferroptotic signaling, neuronal metabolism, and neuroinflammation.

Ferroptosis is a regulated form of cell death driven by iron-dependent lipid peroxidation and oxidative stress (Dixon et al., 2012; Stockwell et al., 2017; Yang and Stockwell, 2016; Li et al., 2020). Accumulating evidence suggests that iron dyshomeostasis plays a key role in AD, particularly in vulnerable brain regions such as the hippocampus and cortex, where excess iron deposition correlates with neuronal loss and cognitive impairment (Wang et al., 2022; Raven et al., 2013; Tran et al., 2022; Bao et al., 2021; Belaidi and Bush, 2016; Ashraf et al., 2018). Consistent with these observations, our transcriptomic analyses revealed significant enrichment of ferroptosis-related pathways in AD brain tissues, supporting the hypothesis that ferroptotic vulnerability represents an essential component of AD pathobiology (Wang et al., 2022; Ma et al., 2022; Zhong et al., 2024). The dysregulation of genes involved in lipid metabolism and redox homeostasis further underscores the relevance of ferroptosis as a driver of neuronal degeneration under chronic oxidative stress.

Beyond intrinsic neuronal susceptibility, ferroptosis may influence AD progression through modulation of the brain immune microenvironment. Neuroinflammation is a well-established hallmark of AD and plays a crucial role in disease initiation and progression (Heneka et al., 2015; De Strooper and Karran, 2016). Our immune infiltration analysis demonstrated significant associations between ferroptosis-related genes and alterations in immune cell populations, suggesting that ferroptotic signaling may actively shape neuroimmune interactions. This is consistent with emerging evidence indicating that ferroptosis can regulate immune responses through lipid peroxidation–derived danger signals and inflammatory mediators (Friedmann Angeli et al., 2019; Wu et al., 2024), thereby creating a feed-forward loop between oxidative injury and chronic inflammation in neurodegenerative conditions.

Through protein–protein interaction network analysis, PPARG emerged as a central hub among ferroptosis-associated genes. PPARG is a nuclear receptor that plays a pivotal role in lipid metabolism, mitochondrial function, and inflammatory regulation (Berger and Moller, 2002; Evans and Mangelsdorf, 2014; Lehrke and Lazar, 2005; Ahmadian et al., 2013). Previous studies have demonstrated that PPARG activation exerts anti-inflammatory and neuroprotective effects in various neurological contexts, including experimental models of AD (Kapadia et al., 2008; Heneka et al., 2005). Our scRNA-seq further revealed that PPARG expression is predominantly enriched in neuronal populations, consistent with previous single-cell studies highlighting cell-type–specific transcriptional remodeling in AD brains (Mathys et al., 2019; Grubman et al., 2019; Leng et al., 2021). These findings provide a cellular basis for the involvement of PPARG in neuronal metabolic adaptation and stress responses.

Notably, although PPARG has been reported as a suppressor of ferroptosis in certain biological contexts (Stockwell et al., 2017; Yang and Stockwell, 2016), our findings indicate a more nuanced, context-dependent role in AD. We observed elevated PPARG expression in AD tissues, which may reflect a compensatory response to chronic oxidative and metabolic stress. However, sustained PPARG activation in a pathological environment characterized by iron overload and lipid peroxidation may become maladaptive. In support of this hypothesis, pharmacological inhibition of PPARG significantly reduced lipid peroxidation, restored redox balance, and attenuated ferroptosis-induced neuronal injury *in vitro*. These results suggest that PPARG function in neurodegeneration is highly dependent on cellular context, ligand availability, and downstream metabolic state.

The therapeutic relevance of PPARG modulation was further supported by our *in vivo* findings. Inhibition of PPARG in an AD-like mouse model improved cognitive performance and alleviated hippocampal neuronal damage, accompanied by reductions in oxidative stress and lipid peroxidation markers. These observations align with growing evidence that targeting ferroptosis-related pathways can confer neuroprotection in AD and other neurodegenerative disorders (Ma et al., 2022; Bao et al., 2021; Zhong et al., 2024). Importantly, our results suggest that selective modulation, rather than broad activation, of PPARG signaling may be required to achieve therapeutic benefit in the context of ferroptosis-driven neurodegeneration.

Several limitations of this study should be acknowledged. First, the D-galactose–induced model primarily reflects aging-associated oxidative stress and does not fully recapitulate the complex amyloid and tau pathology of human AD. Nevertheless, this model has been widely used to investigate oxidative injury and cognitive dysfunction relevant to AD (Ashraf et al., 2018). Future studies employing transgenic AD models or human induced pluripotent stem cell–derived neurons would further strengthen the translational relevance of our findings. Second, although pharmacological inhibition of PPARG produced robust neuroprotective effects, genetic approaches such as neuron-specific knockdown or overexpression will be essential to confirm causality and exclude potential off-target effects. Finally, the interplay between ferroptosis and other regulated cell death pathways, including apoptosis and autophagy, remains to be fully elucidated in the context of AD (Yapryntseva et al., 2022; Su et al., 2019).

FerrDb annotations are curated from studies conducted in varied cell types and conditions; thus, “driver/suppressor” labels should be viewed as context-dependent rather than universal. While PPARG is annotated as a ferroptosis suppressor in some settings, our neuronal experiments show that PPARG inhibition reduces lipid peroxidation and ferroptosis-associated phenotypes, suggesting that in neurons under AD-relevant stress, PPARG signaling may contribute to lipid remodeling that heightens ferroptotic susceptibility.

## Limitations of immune deconvolution in brain tissue

Immune “fractions” inferred from bulk brain transcriptomes should be interpreted cautiously. CIBERSORT results depend on the chosen signature matrix (e.g., LM22), which was developed largely from peripheral immune cells and may not fully represent brain-resident microglial activation states. In addition, AD brain tissues exhibit marked shifts in neuronal and glial composition, which can confound bulk expression-based deconvolution. Therefore, we interpret the observed increase in M2-like myeloid signatures as relative enrichment signals rather than direct evidence of peripheral macrophage infiltration. To improve robustness, we cross-validated findings using brain-relevant marker scoring and/or scRNA-seq–guided deconvolution.

This study integrates bulk and single-cell transcriptomics, network analysis, immune deconvolution, and experimental validation. While the transcriptomic and network-based analyses identify robust associations between PPARG, ferroptosis-related pathways, and immune signatures, they are inherently correlative and cannot independently establish causality. Causal inference in this study is therefore derived primarily from experimental perturbation of PPARG signaling using pharmacological inhibition and corresponding ferroptosis readouts. Additional genetic and longitudinal studies will be required to fully define causal relationships in human Alzheimer’s disease.

The D-galactose model employed in this study primarily reflects aging-associated oxidative stress, neuroinflammation, and neurodegeneration, and does not fully recapitulate the hallmark amyloid-β or tau pathology observed in transgenic Alzheimer’s disease models. Therefore, our *in vivo* findings should be interpreted as evidence supporting a role of PPARG-mediated ferroptosis in neurodegenerative and neuroinflammatory processes, rather than definitive Alzheimer’s disease–specific mechanisms. Validation in transgenic AD models will be an important direction for future studies.

## Conclusion

This study demonstrates that ferroptosis is a key contributor to Alzheimer’s disease pathogenesis and identifies PPARG as a central regulator linking lipid metabolism, iron dysregulation, and neuronal vulnerability. Integrated transcriptomic, single-cell, and experimental analyses reveal neuron-specific PPARG dysregulation and show that PPARG inhibition effectively suppresses ferroptotic damage both *in vitro* and *in vivo*. These findings highlight PPARG-mediated ferroptosis as a promising therapeutic target for mitigating neurodegeneration in Alzheimer’s disease.

## Data Availability

The original contributions presented in the study are included in the article/Supplementary material, further inquiries can be directed to the corresponding author.

## Footnotes

1
http://www.zhounan.org/ferrdb/current/

2
https://cn.string-db.org/

## References

[ref1] Ahmadian M. Suh J. M. Hah N. Liddle C. Atkins A. R. Downes M. . (2013). PPARγ signaling and metabolism: the good, the bad and the future. Nat. Med. 19, 557–566. doi: 10.1038/nm.3159, 23652116 PMC3870016

[ref2] Ashraf A. Clark M. So P. W. (2018). The aging of iron man. Front. Aging Neurosci. 10:65. doi: 10.3389/fnagi.2018.00065, 29593525 PMC5857593

[ref3] Bao W. D. Pang P. Zhou X. T. Hu F. Xiong W. Chen K. . (2021). Loss of ferroportin induces memory impairment by promoting ferroptosis in Alzheimer’s disease. Cell Death Differ. 28, 1548–1562. doi: 10.1038/s41418-020-00685-9, 33398092 PMC8166828

[ref4] Belaidi A. A. Bush A. I. (2016). Iron neurochemistry in Alzheimer's disease and Parkinson's disease: targets for therapeutics. J. Neurochem. 139, 179–197. doi: 10.1111/jnc.13425 26545340

[ref5] Berger J. Moller D. E. (2002). The mechanisms of action of PPARs. Annu. Rev. Med. 53, 409–435. doi: 10.1146/annurev.med.53.082901.104018, 11818483

[ref6] Cummings J. Zhou Y. Lee G. Zhong K. Fonseca J. Cheng F. (2024). Alzheimer's disease drug development pipeline: 2024. Alzheimers Dement. (N. Y.) 10:e12465. doi: 10.1002/trc2.12465, 38659717 PMC11040692

[ref7] De Strooper B. Karran E. (2016). The cellular phase of Alzheimer’s disease. Cell 164, 603–615. doi: 10.1016/j.cell.2015.12.056 26871627

[ref8] Dixon S. J. Lemberg K. M. Lamprecht M. R. Skouta R. Zaitsev E. M. Gleason C. E. . (2012). Ferroptosis: an iron-dependent form of nonapoptotic cell death. Cell 149, 1060–1072. doi: 10.1016/j.cell.2012.03.042, 22632970 PMC3367386

[ref9] Do Van B. Gouel F. Jonneaux A. Timmerman K. Gelé P. Pétrault M. . (2016). Ferroptosis, a newly characterized form of cell death in Parkinson's disease that is regulated by PKC. Neurobiol. Dis. 94, 169–178. doi: 10.1016/j.nbd.2016.05.011, 27189756

[ref10] Evans R. M. Mangelsdorf D. J. (2014). Nuclear receptors, RXR, and the big bang. Cell 157, 255–266. doi: 10.1016/j.cell.2014.03.012, 24679540 PMC4029515

[ref11] Friedmann Angeli J. P. Krysko D. V. Conrad M. (2019). Ferroptosis at the crossroads of cancer-acquired drug resistance and immune evasion. Nat. Rev. Cancer 19, 405–414. doi: 10.1038/s41568-019-0149-1, 31101865

[ref12] Grubman A. Chew G. Ouyang J. F. Sun G. Choo X. Y. McLean C. . (2019). A single-cell atlas of entorhinal cortex from individuals with Alzheimer’s disease reveals cell-type-specific gene expression regulation. Nat. Neurosci. 22, 2087–2097. doi: 10.1038/s41593-019-0539-4, 31768052

[ref13] Heneka M. T. Carson M. J. El Khoury J. Landreth G. E. Brosseron F. Feinstein D. L. . (2015). Neuroinflammation in Alzheimer's disease. Lancet Neurol. 14, 388–405. doi: 10.1016/s1474-4422(15)70016-5, 25792098 PMC5909703

[ref14] Heneka M. T. Sastre M. Dumitrescu-Ozimek L. Hanke A. Dewachter I. Kuiperi C. . (2005). Acute treatment with the PPARγ agonist pioglitazone and ibuprofen reduces glial inflammation and Aβ1–42 levels in APPV717I transgenic mice. Brain 128, 1442–1453. doi: 10.1093/brain/awh452, 15817521

[ref15] Kapadia R. Yi J. H. Vemuganti R. (2008). Mechanisms of anti-inflammatory and neuroprotective actions of PPAR-gamma agonists. Front. Biosci. (Landmark Ed) 13:1813. doi: 10.2741/2802 PMC2734868 17981670

[ref16] Lane C. A. Hardy J. Schott J. M. (2018). Alzheimer's disease. Eur. J. Neurol. 25, 59–70. doi: 10.1111/ene.13439 28872215

[ref17] Lehrke M. Lazar M. A. (2005). The many faces of PPARγ. Cell 123, 993–999. doi: 10.1016/j.cell.2005.11.026, 16360030

[ref18] Leng K. Li E. Eser R. Piergies A. Sit R. Tan M. . (2021). Molecular characterization of selectively vulnerable neurons in Alzheimer’s disease. Nat. Neurosci. 24, 276–287. doi: 10.1038/s41593-020-00764-7, 33432193 PMC7854528

[ref19] Li J. Cao F. Yin H. L. Huang Z. J. Lin Z. T. Mao N. . (2020). Ferroptosis: past, present and future. Cell Death Dis. 11:88. doi: 10.1038/s41419-020-2298-2, 32015325 PMC6997353

[ref20] Li P. Yu J. Huang F. Zhu Y. Y. Chen D. D. Zhang Z. X. . (2023). SLC7A11-associated ferroptosis in acute injury diseases: mechanisms and strategies. Eur. Rev. Med. Pharmacol. Sci. 27, 4386–4398. doi: 10.26355/eurrev_202305_32444 37259719

[ref21] Long J. M. Holtzman D. M. (2019). Alzheimer disease: an update on pathobiology and treatment strategies. Cell 179, 312–339. doi: 10.1016/j.cell.2019.09.001, 31564456 PMC6778042

[ref22] Ma H. Dong Y. Chu Y. Guo Y. Li L. (2022). The mechanisms of ferroptosis and its role in Alzheimer’s disease. Front. Mol. Biosci. 9:965064. doi: 10.3389/fmolb.2022.965064, 36090039 PMC9459389

[ref23] Mathys H. Davila-Velderrain J. Peng Z. Gao F. Mohammadi S. Young J. Z. . (2019). Single-cell transcriptomic analysis of Alzheimer’s disease. Nature 570, 332–337. doi: 10.1038/s41586-019-1195-2, 31042697 PMC6865822

[ref24] Raven E. P. Lu P. H. Tishler T. A. Heydari P. Bartzokis G. (2013). Increased iron levels and decreased tissue integrity in hippocampus of Alzheimer's disease detected *in vivo* with magnetic resonance imaging. J. Alzheimer's Dis 37, 127–136. doi: 10.3233/jad-130209, 23792695

[ref25] Selkoe D. J. Hardy J. (2016). The amyloid hypothesis of Alzheimer's disease at 25 years. EMBO Mol. Med. 8, 595–608. doi: 10.15252/emmm.201606210, 27025652 PMC4888851

[ref26] Stockwell B. R. Angeli J. P. F. Bayir H. Bush A. I. Conrad M. Dixon S. J. . (2017). Ferroptosis: a regulated cell death nexus linking metabolism, redox biology, and disease. Cell 171, 273–285. doi: 10.1016/j.cell.2017.09.021, 28985560 PMC5685180

[ref27] Su L. J. Zhang J. H. Gomez H. Murugan R. Hong X. Xu D. . (2019). Reactive oxygen species-induced lipid peroxidation in apoptosis, autophagy, and ferroptosis. Oxidative Med. Cell. Longev. 2019:5080843. doi: 10.1155/2019/5080843 PMC6815535 31737171

[ref28] Tran D. DiGiacomo P. Born D. E. Georgiadis M. Zeineh M. (2022). Iron and Alzheimer’s disease: from pathology to imaging. Front. Hum. Neurosci. 16:838692. doi: 10.3389/fnhum.2022.838692, 35911597 PMC9327617

[ref29] Wang F. Wang J. Shen Y. Li H. Rausch W. D. Huang X. (2022). Iron dyshomeostasis and ferroptosis: a new Alzheimer’s disease hypothesis? Front. Aging Neurosci. 14:830569. doi: 10.3389/fnagi.2022.830569 35391749 PMC8981915

[ref30] Wu J. Li Z. Wu Y. Cui N. (2024). The crosstalk between exosomes and ferroptosis: a review. Cell Death Discov. 10:170. doi: 10.1038/s41420-024-01938-z, 38594265 PMC11004161

[ref31] Yang W. S. Stockwell B. R. (2016). Ferroptosis: death by lipid peroxidation. Trends Cell Biol. 26, 165–176. doi: 10.1016/j.tcb.2015.10.014, 26653790 PMC4764384

[ref32] Yapryntseva M. A. Maximchik P. V. Zhivotovsky B. Gogvadze V. (2022). Mitochondrial sirtuin 3 and various cell death modalities. Front. Cell Dev. Biol. 10:947357. doi: 10.3389/fcell.2022.947357, 35938164 PMC9354933

[ref33] Zeisel J. Bennett K. Fleming R. (2020). World Alzheimer Report 2020: Design, Dignity, Dementia: Dementia-Related design and the Built Environment.

[ref34] Zhong H. Liu H. Fu Q. (2024). Ferroptosis as a therapeutic target in neurodegenerative diseases: exploring the mechanisms and potential of treating Alzheimer's disease and Parkinson's disease. Protein Pept. Lett. 31, 759–772. doi: 10.2174/0109298665333926240927074528, 39513303

